# LoRaWAN Transmissions in Salt Water for Superficial Marine Sensor Networking: Laboratory and Field Tests

**DOI:** 10.3390/s23104726

**Published:** 2023-05-13

**Authors:** Alessandro Pozzebon, Irene Cappelli, Filippo Campagnaro, Roberto Francescon, Michele Zorzi

**Affiliations:** 1 Department of Information Engineering, University of Padova, Via Gradenigo 6/b, 35131 Padova, Italy; alessandro.pozzebon@unipd.it (A.P.); filippo.campagnaro@unipd.it (F.C.); frances1@dei.unipd.it (R.F.); zorzi@dei.unipd.it (M.Z.); 2 Department of Information Engineering and Mathematics, University of Siena, Via Roma 56, 53100 Siena, Italy

**Keywords:** Internet of Underwater Things, LoRa, LoRaWAN, marine sensor networks, salt water, transmission performance, underwater to above water

## Abstract

In this paper, the authors present the results of a set of measurements carried out to analyze the transmission capabilities of the LoRaWAN technology for underwater to above water transmission in saline water. A theoretical analysis was used to model the link budget of the radio channel in the considered operative conditions and to estimate the electrical permittivity of salt water. Preliminary measurements were performed in the laboratory at different salinity levels to confirm the application boundaries of the technology, then field tests were conducted in the Venice lagoon. While these test are not focused on demonstrating the usability of LoRaWAN to collect data underwater, the achieved results demonstrate that LoRaWAN transmitters can be used in all those conditions when they are expected to be partially or totally submerged below a thin layer of marine water, in accordance with the prediction of the proposed theoretical model. This achievement paves the way for the deployment of superficial marine sensor networks in the Internet of Underwater Things (IoUT) context, as for the monitoring of bridges, harbor structures, water parameters and water sport athletes and for the realization of high-water or fill-level alarm systems.

## 1. Introduction

Data transmission in the presence of water is a well-known problem that limits the adoption of wirelessly connected devices in a plethora of applications. The effect of water not only limits the deployment of underwater devices, but also poses strict limitations on the positioning of data transmission tools in places where there is a significant presence of water: this is the case for example of floating structures, where antennas have to be deployed at a certain height above water to ensure successful transmissions.

Due to the importance of transmitting data from under water, huge efforts have been put in the last years to identify suitable solutions: however, these are mainly based on transmission channels that do not exploit the electromagnetic (EM) waves as transmission media. As a matter of fact, the most effective solutions are currently based on acoustic and optical signals [1,2]. However, obviously both these techniques have strong limitation that hinder their adoption. Indeed, while acoustic waves notably suffer for the presence of environmental noise, optical signals are affected by the presence of turbidity and sun light. EM waves, which in principle overcome these limitations, are severely attenuated by water, due to its conductive behavior, and this notably limits the achievable transmission range. This is even more significant in salt water, where the presence of dissolved salts increases the conductivity, raising also the attenuation. The transmission range is also affected by the frequency of the radio wave, with an inverse relation: the higher the frequency, the shorter the transmission range. As a matter of fact, effective radio transmission in water can be achieved only in Extremely Low Frequency (ELF) and Very Low Frequency (VLF) bands, which feature significant technical limitations preventing the realization of integrated data transmission modules. The most part of portable communication technologies relies on notably higher frequencies, in the Ultra High Frequency (UHF) and Microwave bands: at these frequencies, the implementation of an underwater transmission channel appears to be totally infeasible.

Even if a long range underwater radio transmission using standard communication technologies is out of target, the chance to transmit data from limited depths (below 1 m) may be important for a number of applications, in particular, wireless sensor systems in water: some examples include the monitoring of bridges and harbor structures, the acquisition of water parameters or the monitoring of water sport athletes. In all these cases, sensors may be deployed at limited depths, with the chance of transmitting data directly from under water, without the need of installing ad hoc structures to position the transmitting antenna outside the water, possibly at a certain height.

With the development of the Internet of Things (IoT) concept, a number of radio technologies have emerged in the last 15 years with the aim of providing efficient data transmission in terms of wide coverage and reduced power consumption. Most of them operate in the unlicensed Industrial, Scientific and Medical (ISM) bands at the frequencies of 433 MHz, 868 MHz and 915 MHz, and exploit a number of techniques to increase the overall transmission efficiency. Among all these technologies, LoRa (acronym of Long Range) [3] has rapidly gained momentum thanks to its excellent features in terms of receiver sensitivity, which allow it to achieve very long transmission ranges at the price of a limited power consumption. Thanks to the Chirp Spread Spectrum (CSS) modulation, receiver sensitivity values up to −148 dBm are achieved, which corresponds to transmission ranges up to some kms in urban centers and some tens of kms in rural areas. Together with LoRa, the MAC layer LoRaWAN (LoRa Wide Area Network) protocol allows the implementation of complex network architectures integrating thousands of nodes.

Thanks to its excellent features, LoRaWAN has been identified as an ideal candidate for all those applications where critical environmental conditions limit the operation of other competing technologies. Indeed, LoRaWAN has proven to be effective for deploying underground to above ground transmission channels [4], as well as in the presence of metallic housings [5] and in severe industrial contexts [6]. Tests were performed also to analyze the performance for transmissions from under to above water in fresh water [7]: feasibility was demonstrated for transmitter depths up to more than 1 m, suggesting the usability of this technology for a number of applications [8].

The aim of this work is to test the performance of LoRaWAN for underwater to above water transmissions with the transmitter positioned in salt water at first by means of a theoretical analysis, then by means of preliminary laboratory measurements for different salinity levels and at different depths. Finally, a set of qualitative tests was carried out in sea water.

The rest of the paper is structured as follows: Section 2 reviews the scientific contributions concerning radio transmission from salt water; Section 3 describes the theoretical analysis of EM propagation through a lossy medium, focusing on the estimation of the complex permittivity of salt water and on the mathematical modeling of link budget and path loss in underwater to above water transmissions; Section 4 illustrates the experimental setup adopted during the tests; Section 5 presents and discusses the experimental outcomes of the performed measurement campaigns; finally, in Section 6 the conclusions are provided.

## 2. Related Works

The chance of exploiting radio transmission in salt water was analyzed more than 50 years ago [9,10,11], underlining the severe attenuation that limited the usage of this technology to very few specific contexts. While the limited chances of exploiting EM waves for data transmission in salt water are well known, in the last years a number of application contexts emerged, where radio technologies may be exploited within the marine environment. Platforms like unmanned surface vehicles, water quality monitoring stations or structural health monitoring systems for infrastructures like harbors or bridges may exploit the radio channel for the transmission of collected data. Indeed, in several cases data is not required to be transmitted from high depths: for this reason, in the last years a limited number of contributions have emerged in the literature trying to analyze the usability of radio technologies in the presence of salt water. In this context, the Wireless Sensor Networks (WSNs) can be a promising tool for monitoring marine environments thanks to its strengths as the quasi real-time data transmission, the pervasive deployment, the scalability, the relatively low-cost and the unmanned operations [12]. In this regard, a new term has been coined to identify the IoT systems designed for underwater applications, the Internet of Underwater Things (IoUT).

While a set of works focuses on the implementation of transmitters operating in the ELF to VLF bands [13], some others analyse the realization of tools operating at higher frequencies. Significant results were achieved in the High Frequency (HF) band: in [14] transmission in salt water was successfully achieved on field at a 1 MHz frequency, while in [15] frequencies up to 5 MHz were tested at distances up to 90 m. One of the techniques that have been employed to set up radio channels between partially submerged transmitters or receivers is the exploitation of lateral waves [16,17]. In this case, short range successful transmission was also achieved at higher frequencies: in particular, in [17] also the 2.4 GHz band was tested, suggesting very short range transmission with common technologies like WiFi. An ad hoc antenna, to be employed under sea water to transmit at 2.4 GHz was also presented in [18]: 2.4 GHz transmission was also tested respectively in pools with commercial XBee modules in [19] and implementing ZigBee protocol in [20].

Despite these contributions, there is currently in the literature a lack of works analyzing the behavior in salt water of data transmission technologies operating at the so-called Sub-GHz frequencies, i.e., those frequencies below 1 GHz. These include a number of technologies like SigFox or NB-IoT that are playing a significant role in the spreading of IoT solutions. Due to its large diffusion, LoRa is the only technology whose usability in water has been partially analyzed. However, contributions only deal with fresh water [7,8,21,22], with a single work proposing some tests in salt water [23]: nevertheless, these were carried out exploiting an ad hoc antenna and thus not setting up an actual LoRaWAN transmission channel. Therefore, to the best of our knowledge, this is the first work discussing the behavior of LoRaWAN technology in salt water.

Demonstrating the applicability of these technologies to the marine context can facilitate the deployment of WSNs in manifold scenarios of interest for the scientific community, and in several cases quasi-superficial data transmissions are sufficient for the required task. It is the case of the structural health monitoring of bridges and harbors [24,25], the supervising of the vital parameters of athletes during water sport training [26] and the monitoring of fish farms [27]. However, the presence of the human activities also has indirect implications in the modification of the aquatic habitats, for this reason other relevant research topics are those aimed at the monitoring of water quality [28], water pollution [29] and coral reef ecosystems [30]. Finally, even the realization of high-water or fill-level alarm systems can be a topic of interest in those scenarios where the timely communication of the water level rise is crucial, e.g., in case of tides [31].

## 3. Theoretical Analysis

In this section, the theoretical background behind the mathematical modeling of the underwater to above water radio channel is reviewed. In particular, the physical laws ruling the propagation of a generic EM signal in a lossy medium are investigated, limiting the derivation of the complex relative permittivity to the saline water case, which is a high-loss medium due to the presence of dissolved salts that increase the medium conductivity; finally, the modeling of the link budget and of the path loss is presented.

### 3.1. Electromagnetic Propagation

The propagation of an EM wave in a lossy medium is ruled by the complex propagation constant of the medium γ, which reveals how the signal is attenuated during its spreading in space. Its general expression is reported in Equation (1) and is the combination of a real component α, also called attenuation constant and accounting for the wave amplitude decay in 1/m, and an imaginary component β, also called phase constant since it is related to the variations of the wave phase in rad/m [32,33].

(1) $$\gamma =j\omega \sqrt{\mu \tilde{\varepsilon }}=\alpha +j\beta .$$

The unknown terms in Equation (1) are the angular frequency ω=2πf, with *f* the frequency of the propagating signal, the permittivity $\tilde{\varepsilon }$ and the magnetic permeability $\mu =\mu_{0}\mu_{r}=4\pi \times 10^{-7}$ H/m $\times \mu_{r}=1.2566\times 10^{-6}$ H/m $\times \mu_{r}$, with $\mu_{0}$ the permeability of vacuum and $\mu_{r}$ the permeability of the medium ($\mu_{r}=$ 1 in case of salt water since it is a non-magnetic material). Concerning the permittivity, it is a complex number depending on the conductivity, the dielectric constant of the medium and the frequency of the propagating signal, and influenced by some other parameters such as the temperature [34]. It can be defined as

(2) $$\tilde{\varepsilon }=\varepsilon_{0}\tilde{\varepsilon_{r}}=\varepsilon_{0}\left(\varepsilon_{r}^{\prime }-j\varepsilon_{r}^{\prime \prime }\right)=\varepsilon -j\frac{\sigma }{\omega }$$

with $\varepsilon_{0}=8.85\times 10^{-12}$ F/m the permittivity of vacuum and σ the electrical conductivity of the medium in S/m, $\tilde{\varepsilon_{r}}=\varepsilon_{r}^{\prime }-j\varepsilon_{r}^{\prime \prime }$ is the complex relative permittivity of the medium. Consequently Equation (1) becomes

(3) $$\gamma =j\omega \sqrt{\mu \tilde{\varepsilon }}=j\omega \sqrt{\mu \varepsilon (1-j\frac{\sigma }{\omega \varepsilon })}=\alpha +j\beta .$$

The constants α and β can be derived according to the following formulas [32,33,35]:

(4) $$\alpha =\omega \sqrt{\frac{\mu \varepsilon }{2}\left(\sqrt{1+{\left(\frac{\sigma }{\omega \varepsilon }\right)}^{2}}-1\right)}$$

(5) $$\beta =\omega \sqrt{\frac{\mu \varepsilon }{2}\left(\sqrt{1+{\left(\frac{\sigma }{\omega \varepsilon }\right)}^{2}}+1\right)}.$$

When the EM wave propagates across two different media ($m_{1}$ and $m_{2}$), the phenomena of reflection and refraction at their interface must be accounted for. In case of normal incidence, the expressions of reflection and transmission coefficients simplify becoming [36]

(6) $$\Gamma =\frac{\eta_{m_{2}}-\eta_{m_{1}}}{\eta_{m_{2}}+\eta_{m_{1}}}$$

(7) $$\tau =\frac{2\eta_{m_{2}}}{\eta_{m_{2}}+\eta_{m_{1}}}$$

where $\eta_{m_{2}}$ and $\eta_{m_{1}}$ are the complex intrinsic impedances of the media in Ω [36] computed as

(8) $$\eta =\sqrt{\frac{j\omega \mu }{\left(j\omega \varepsilon +\sigma \right)}}.$$

### 3.2. Complex Relative Permittivity

The complex relative permittivity $\tilde{\varepsilon_{r}}$ of the saline water is derived according to the model proposed in [37] by the International Telecommunication Union (ITU) and is valid for evaluating the effect of salinity on radio transmission [38]; in particular, the real and imaginary parts of $\tilde{\varepsilon_{r}}$ are computed through the following formulas:

(9) $$\varepsilon_{r}^{\prime }=\frac{\varepsilon_{ss}-\varepsilon_{1s}}{1+{\left(\frac{f_{GHz}}{f_{1s}}\right)}^{2}}+\frac{\varepsilon_{1s}-\varepsilon_{\infty s}}{1+{\left(\frac{f_{GHz}}{f_{2s}}\right)}^{2}}+\varepsilon_{\infty s}$$

(10) $$\varepsilon_{r}^{\prime \prime }=\frac{\left(\frac{f_{GHz}}{f_{1s}}\right)\left(\varepsilon_{ss}-\varepsilon_{1s}\right)}{1+{\left(\frac{f_{GHz}}{f_{1s}}\right)}^{2}}+\frac{\left(\frac{f_{GHz}}{f_{2s}}\right)\left(\varepsilon_{1s}-\varepsilon_{\infty s}\right)}{1+{\left(\frac{f_{GHz}}{f_{2s}}\right)}^{2}}+\frac{18\sigma_{r}}{f_{GHz}}$$

where $f_{GHz}$ is the working frequency in GHz and $\varepsilon_{ss},\varepsilon_{1s},\varepsilon_{\infty s},f_{1s},f_{2s}$ are derived as

(11) $$\varepsilon_{ss}=\varepsilon_{s}e^{-3.3333\times 10^{-3}S+4.74868\times 10^{-6}S^{2}}$$

(12) $$\varepsilon_{1s}=\varepsilon_{1}e^{-6.28908\times 10^{-3}S+1.76032\times 10^{-4}S^{2}-9.22144\times 10^{-5}TS}$$

(13) $$\varepsilon_{\infty s}=\varepsilon_{\infty }\left(1+S\left(-2.04265\times 10^{-3}+1.57883\times 10^{-4}T\right)\right)$$

(14) $$\begin{array}{c} f_{1s}=f_{1}(1+S(2.3232\times 10^{-3}-7.9208\times 10^{-5}T+3.6764\times 10^{-6}T^{2}+\\ 3.5594\times 10^{-7}T^{3}+8.9795\times 10^{-9}T^{4}))\;\mathrm{GHz} \end{array}$$

(15) $$f_{2s}=f_{2}\left(1+S\left(-1.99723\times 10^{-2}+1.81176\times 10^{-4}T\right)\right)\;\mathrm{GHz}.$$

In the previous equations, *S* is the salinity (in g/L or ppt) and T is the operating temperature in Celsius degrees, while the dimensionless terms $\varepsilon_{1},\varepsilon_{s},\varepsilon_{\infty }$ and the Debye relaxation frequencies $f_{1},f_{2}$ are calculated as

(16) $$\varepsilon_{s}=77.66+103.3\Theta$$

(17) $$\varepsilon_{1}=0.0671\varepsilon_{s}$$

(18) $$\varepsilon_{\infty }=3.52-7.52\Theta$$

(19) $$f_{1}=20.20-146.4\Theta +316\Theta^{2}\;\mathrm{GHz}$$

(20) $$f_{2}=39.8f_{1}\;\mathrm{GHz}$$

with $\Theta =\frac{300}{T+273.15}-1$. Moreover, $\sigma_{r}$ is computed from

(21) $$\sigma_{r}=\sigma_{35}R_{15}R_{T15}\;S/m$$

with

(22) $$\begin{array}{c} \sigma_{35}=2.903602+8.607\times 10^{-2}T+4.738817\times 10^{-4}T^{2}-2.991\times 10^{-6}T^{3}\\ +4.3047\times 10^{-9}T^{4} \end{array}$$

(23) $$R_{15}=S\frac{37.5109+5.45216S+1.4409\times 10^{-2}S^{2}}{1004.75+182.283S+S^{2}}$$

(24) $$R_{T15}=1+\frac{\alpha_{0}\left(T-15\right)}{\alpha_{1}+T}$$

(25) $$\alpha_{0}=\frac{6.9431+3.2841S-9.9486\times 10^{-2}S^{2}}{84.850+69.024S+S^{2}}$$

(26) $$\alpha_{1}=49.843-0.2276S+0.198\times 10^{-2}S^{2}.$$

Once $\tilde{\varepsilon_{r}}=\varepsilon_{r}^{\prime }-j\varepsilon_{r}^{\prime \prime }$ is calculated, and knowning the angular frequency ω of the propagating signal and the magnetic permittivity μ, the amplitude and phase constants α and β can be derived according to Equations (4) and (5).

### 3.3. Link Budget

The link budget model describing the propagation of the EM wave is reported in Equation (27): the received power $P_{r}$ in dBm is derived from the transmitted power $P_{t}$ in dBm, the receiving and transmitting antenna gains $G_{r}$ and $G_{t}$ in dBi and the total path loss $PL_{tot}$ in dB which, in the considered application, models the losses of the underwater to above water radio channel, hence assuming $m_{1}$ as saline water and $m_{2}$ as free air [35,36,39].

(27) $$P_{r}=P_{t}+G_{r}+G_{t}-PL_{tot}.$$

In detail, $PL_{tot}$ can be obtained as the summation of the underwater losses, $L_{UW}$, the losses due to the reflections at the water-air interface, $L_{UW-AW}$, and the free space losses, $L_{AW}$. These contributions can be derived as follows:

(28) $$L_{UW}=8.69\alpha d_{UW}+20\log_{10}d_{UW}+20\log_{10}\beta +6$$

(29) $$L_{UW-AW}=10\log_{10}({\left|\tau \right|}^{2}Re\left\{\frac{\eta_{W}}{\eta_{A}}\right\}{)}^{-1}$$

(30) $$L_{AW}=20\log_{10}d_{AW}+20\log_{10}f-147.5$$

where $d_{UW}$ and $d_{AW}$ are the distances (in m) travelled respectively underwater, before reaching the water-air discontinuity, and above water, *f* is the working frequency (in Hz), α and β are the attenuation and the phase constants computed as in Equations (4) and (5), τ is the transmission coefficient at the interface derived according to Equation (7) and $\eta_{A}$ and $\eta_{W}$ are the intrinsic impedances of air and water computed as in Equation (8).

## 4. Experimental Setup

**Figure 1:**
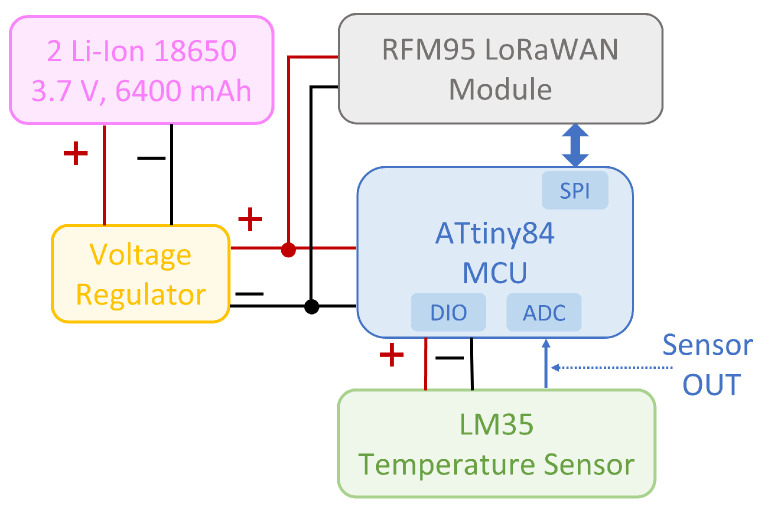
Architecture of the transmitter node.

Two different measurement campaigns were performed, respectively in the laboratory and in the field; in both cases the same ad hoc hardware infrastructure was set up for what concerns transmitting and receiving nodes. In particular, a custom made LoRaWAN end node and an off-the-shelf LG308 Dragino Gateway, featuring a 2 dBi λ/4 antenna and connected to a 4G router for data forwarding to the LoRaWAN server, were exploited. The transmitter node, whose architecture is schematized in Figure 1, was based on an RFM95x LoRa transceiver by HopeRF featuring a λ/8 antenna with 2 dBi gain, integrated with an ATtiny84 microcontroller by Atmel/Microchip. The microcontroller managed the timing and frequency of the transmissions, and collected a sample data from an LM35 temperature sensor: this value was inserted in the default payload for each transmitted packet. The whole transmitting node was powered with the regulated voltage at 3.3 V of two parallel 18650 Li-Ion batteries, with a total capacity of 6400 mAh. Since the node had to be placed under water, all the circuitry was placed inside an ABS IP68 plastic box with dimensions 24 cm × 12 cm × 8 cm: the antenna was fixed to the internal upper surface of the box, oriented perpendicularly with respect to the antenna of the reader.

The transmitter implemented a Class A LoRaWAN End Node: however, to speed up the tests and since the transmission range was notably limited by the water attenuation, the 1% duty-cycle rule was not respected. In order to test the system performance for all the Spreading Factors (SF), an ad hoc firmware was implemented: its operation foresaw the transmission of 300 packets of fixed length for each SF, from 7 to 12, with a payload size of 11 B. The transmitted power was fixed to $P_{t}=$ 14 dBm while the bandwidth (BW), the transmitting frequency *f* and the coding rate (CR) were equal respectively to 125 kHz, 868 MHz and 4/5.

### 4.1. Laboratory Tests

**Figure 2:**
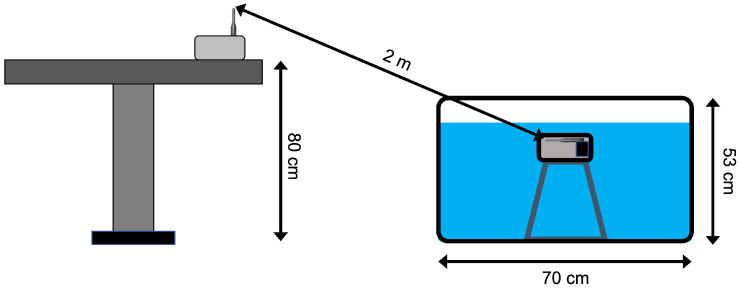
Laboratory test setup.

**Figure 3:**
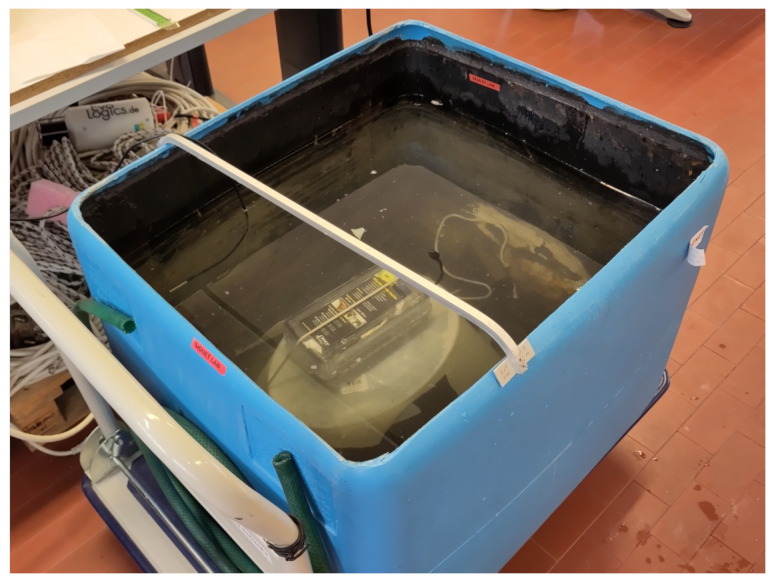
Plastic container used for the laboratory tests.

Laboratory tests were performed exploiting a plastic container filled with 150 L of water, with dimensions 70 cm × 60 cm × 53 cm [40]. These dimensions allowed to have a thinner layer of water above the transmitter box with respect to all the other sides: this aspect was crucial to evaluate the actual transmission performance only as a function of the transmitter depth since the attenuation along the other directions was by far higher than along the perpendicular one. The reader was positioned above a desk near the plastic container, at an approximate height of 1 m. The distance between the reader and the water surface was approximately 2 m. All these distances were measured using a measurement tape with ±0.1 cm of uncertainty: however, due to the difficulty of identifying the exact point above the transmitter in the water surface, a ±0.5 cm uncertainty is assumed. Figure 2 schematizes the experimental setup, while the plastic container with the transmitter can be seen in Figure 3.

Tests were performed positioning the transmitter at two different depths: 6 cm and 12 cm, for both these depths a ±0.5 cm measurement uncertainty can be assumed. This distance was measured from the upper surface of the box, in the spot where the transmitter antenna was fixed. In order to keep the box suspended in the water, it was positioned on a plastic structure and anchored to it using ropes and concrete ballasts.

Together with the tests at different depths, also different salinities for each depth were tested. In particular, since salinity can be expressed in g/L, different levels were achieved by adding increasing amounts of sodium chloride (NaCl) to still water: indeed, NaCl represents around 80% of all dissolved salts. While seawater salinity ranges between 31 g/L and 39 g/L, with a global average value of 35 g/L, lower values were also tested, with the aim of studying LoRaWAN performance also in case of lagoon environments (for example, the Venice lagoon [41]), where salinity can be notably lower than in the open sea. The overall quantity of water was 150 L, thus a 10 g/L salinity could be obtained by dissolving in it 1.5 kg of salt. A total of 9 salinity levels were set up by consequently adding 1.5 kg for the first two levels and then 0.5 kg of salt at a time for the other 7 levels: 0 g/L, 10 g/L, 20 g/L, 23.3 g/L, 26.7 g/L, 30 g/L, 33.3 g/L, 36.7 g/L, 40 g/L and 43.3 g/L. The salt was weighed using a laboratory scale with uncertainty of 2 g. The tested operational conditions are reported in Table 1.

### 4.2. Field Tests

Field tests were performed in Chioggia, Italy: such place is inside the Venice Lagoon, close however to the lagoon mouths and thus featuring a salinity which can be assumed comparable with the one of the northern Adriatic Sea, around 35 g/L [42]. Tests were performed by placing the transmitter node underwater close to the port quay, in order to achieve a distance between the transmitter and the gateway of 2 m, resembling the experimental setup of the laboratory. Such distance was measured again with a measurement tape with ±0.1 cm of uncertainty: however, due to the movements of the water surface such measure can be assumed with a ±5 cm uncertainty.

Concerning the technological setup, the transmitter node, as well as the firmware and the operating principle, was the same employed for the laboratory tests with the only difference that 400 transmissions for each SF from 7 to 12 were performed. Similarly, the same Gateway and 4G dongle were employed: however, since tests were carried out in the field, a 44 Ah, 12 V lead acid battery connected to an inverter was used for powering the system. The gateway was positioned close to the quay edge, in order to ensure direct line of sight with the transmitter.

**Figure 4:**
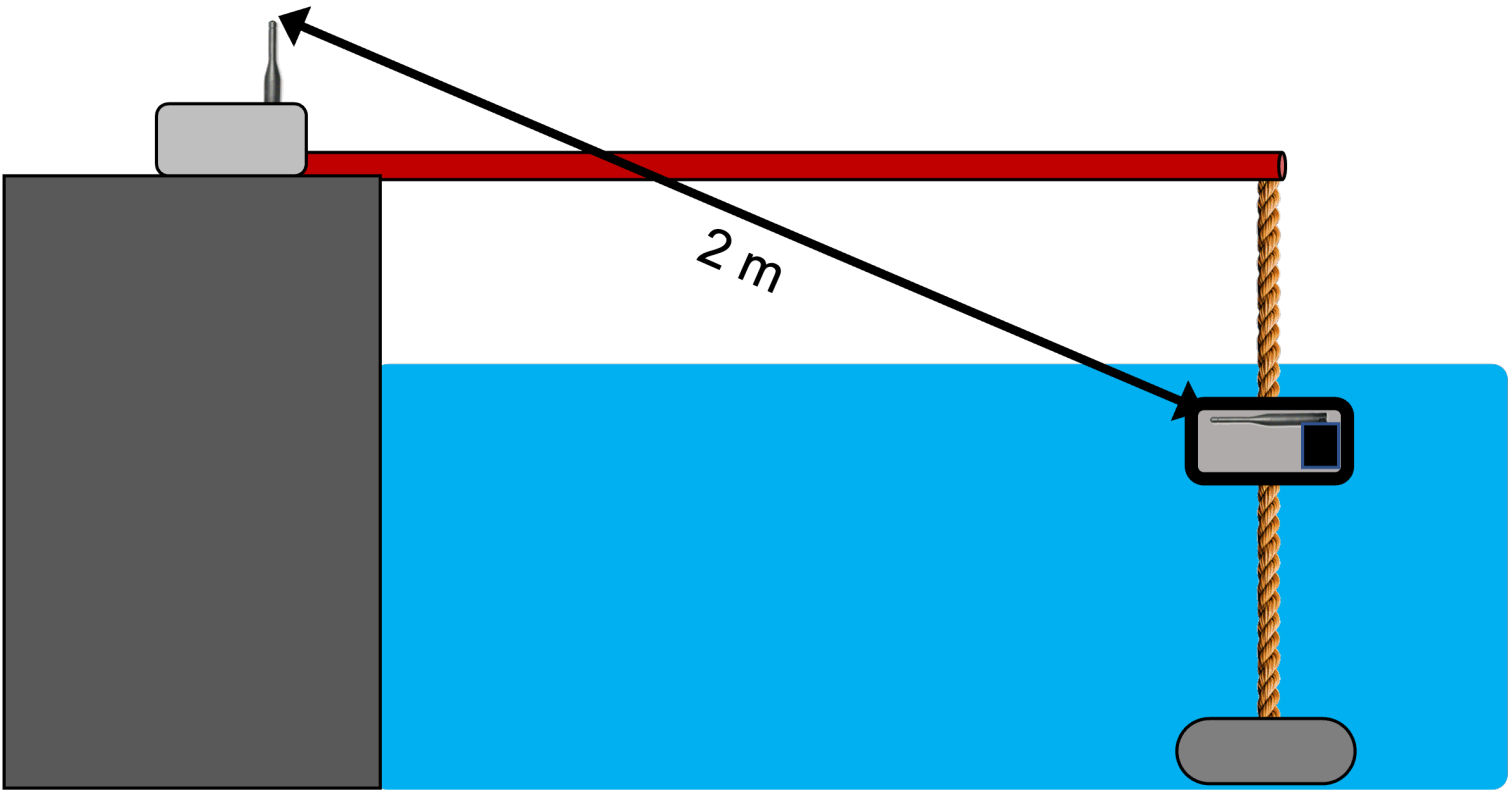
Test setup of the field tests in Chioggia.

**Figure 5:**
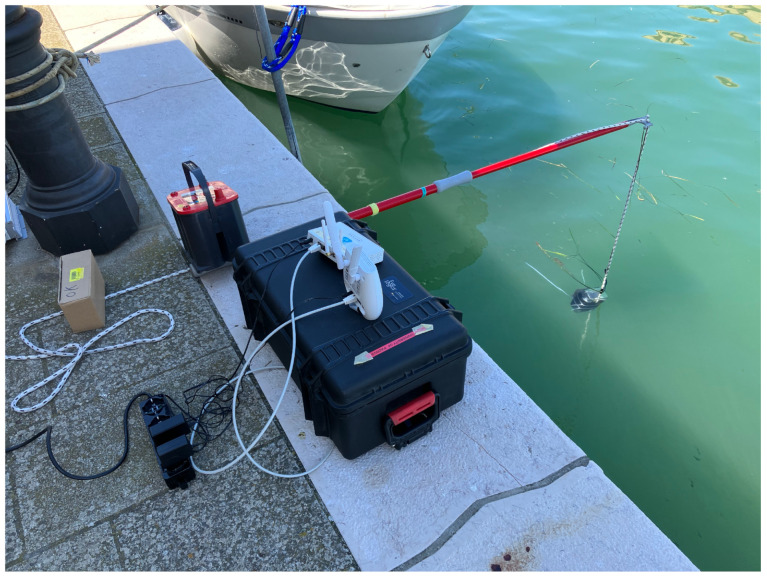
Tests performed in Chioggia harbor.

In order to place the transmitter node under water, at a specific depth and at a distance from the quay which was notably larger than the depth itself to avoid lateral waves, a pole was used. The node was provided with a ballast and was tied to the pole by means of a rope. The overall experimental setup can be seen in Figure 4 while a picture of the tests can be seen in Figure 5.

While for the laboratory tests the positioning of the transmitter node at a specific depth (i.e., 3 cm and 6 cm) was feasible since a flat water surface was easily achievable, this was almost unachievable in the real scenario. Even if the tests were carried out in a sunny day, with a very limited wave motion, some cms of variation have always to be taken into account. Moreover, larger variations were also caused by the passage of boats of different sizes. Together with the actual variations in the sea height, this motion also caused small displacements of the node, which prevented from keeping a constant depth value. For this reason, the results of these tests are to be evaluated mainly from a qualitative point of view rather than from a quantitative one. Due to the difficulty in positioning the node at a specific depth, we can assume the actual depth value to be 6 ± 4 cm. This value is in line with those adopted in the laboratory tests and partially allows a comparison between the two results.

## 5. Results and Discussion

In this Section the results of the two measurement campaigns performed with the setup and the methodology described in Section 4 are presented. The transmission performance of the LoRa technology, implementing the LoRaWAN MAC protocol, is investigated in terms of mean and standard deviation of received signal strength indicator (RSSI) and signal to noise ratio (SNR), and packet loss (PL) percentage. In particular, the SF setting is varied for each tested configuration from 7 to 12 while the other radio settings are left fixed (i.e., CR = 4/5, BW = 125 kHz). This decision was motivated by the fact that, as proved in [8], varying the CR and the BW has no substantial effect in improving the radio performance except for a better error decoding in case of the highest CR, which at the same time leads to higher power consumption. Indeed, the CR setting specifies the ratio between transmitted information bits and redundant parity bits used for forward error correction, hence a lower CR entails more transmitted bits, longer time on air and consequently higher current absorption. On the other side, the SF, which defines the ratio between chip rate and symbol rate, is a relevant parameter for the evaluation of the trade-off between demodulation capability, receiver sensitivity, PL, and power consumption, thus varying the SF in its full range offers a thorough investigation of the LoRa transmission feasibility.

**Figure 6:**
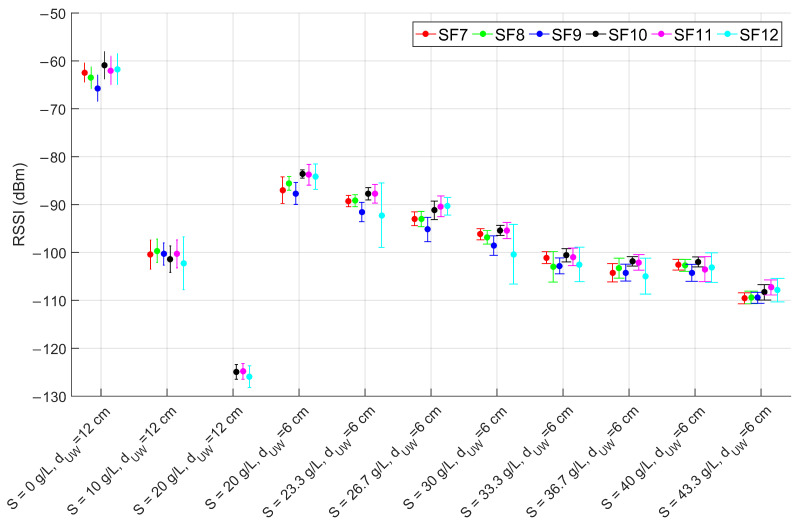
RSSI mean values and standard deviations for SF from 7 to 12 (as per the legend) for the laboratory test configurations reported in Table 1.

**Figure 7:**
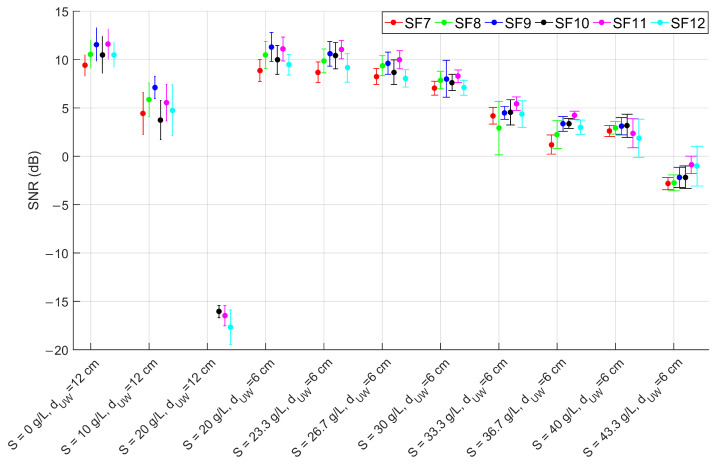
SNR mean values and standard deviations for SF from 7 to 12 (as per the legend) for the laboratory test configurations reported in Table 1.

**Figure 8:**
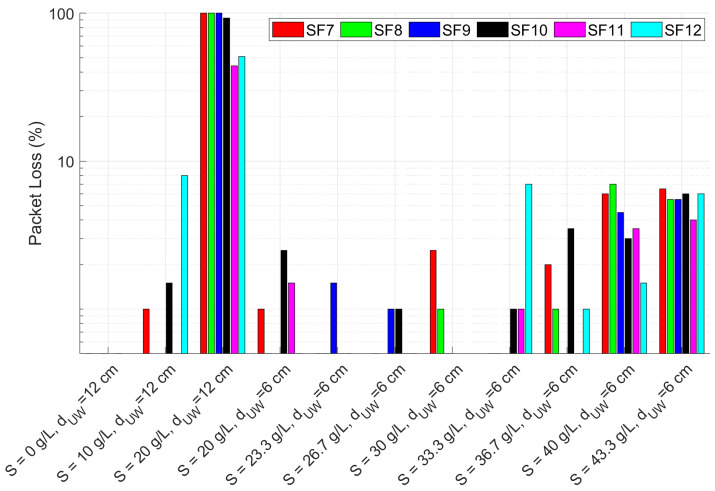
PL percentages for SF from 7 to 12 (as per the legend) for the laboratory test configurations reported in Table 1.

The outcomes of the laboratory tests for the configurations of Table 1 are numerically reported in Table 2 and graphically depicted in Figure 6, Figure 7 and Figure 8. As expected, the change in the SF does not affect the RSSI magnitude but rather the PL, which increases as the SF decreases, in accordance with the LoRa documentation [43]. Moreover, the SNR decreases in conjunction with the increase of the PL, proving the growth of the noise level in the radio channel. In particular, up to a salinity of 30 g/L the PL percentage is consistent with the systematic losses of the radio channel, while for higher salinity the lower SFs start experiencing higher PLs which however, depending on the reliability requirement of the specific application, can still be acceptable. The most critical tested configuration is #3 of Table 1, since the packet reception is hindered for SFs lower than 9 and seriously compromised for the other SFs because of the severe attenuation and the SNR degradation. For this reason we decided not to test lower depths but to increase the salinity fixing the $d_{UW}$ to 6 cm. Considering that the seawater salinity ranges between 31 g/L and 39 g/L, the results for configurations #7–#10 can be assumed representative of realistic deployments in marine water demonstrating that the transmission is feasible only with the receiver submerged by a few cm of water. This limits the use of this technology to almost superficial monitoring applications such as the monitoring of water sport athletes or tides warning systems, or in combination with other techniques with the aim of implementing hybrid networking [44].

**Figure 9:**
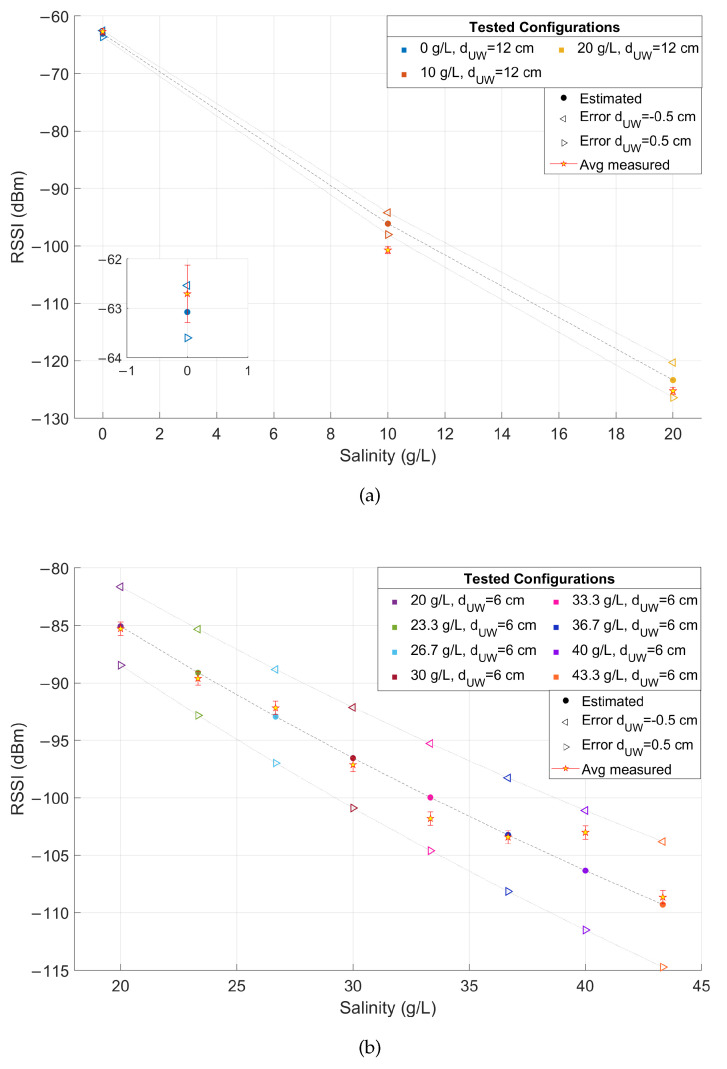
Comparison between modeled RSSI according to Equation (27) (round markers) and measured RSSI during the tests (star markers) as a function of the salinity for (**a**) configurations #1–#3 and (**b**) configurations #4–#11 of Table 1, together with vertical error bars and modeled RSSI assuming a positioning error in $d_{UW}$ of ±0.5 cm (triangular markers). In the inset of (**a**) a magnification of the results for configuration #1 is shown.

These results are also compared with the link budget model proposed in Equation (27), considering that in the examined application $P_{r}$ is the RSSI downstream of the demodulator, $P_{t}=$ 14 dBm, $G_{r}=$ 2 dBi and $G_{t}=$ 2 dBi. Equations (28)–(30) are derived assuming $d_{UW}$ as in Table 1, $d_{AW}=2$ m, f=868 MHz and $\eta_{A}=376.73\;\Omega$. The values of τ, $\eta_{W}$, α and β are computed respectively from Equations (7), (8), (4) and (5) once the complex permittivity is estimated using Equations (9)–(18) (assuming operating temperature *T* = 20 ${}^{{}^{\circ}}$C and the salinity values reported in Table 1). A comparison between model-estimated (round markers) and actually measured (star markers) RSSI is reported in Figure 9 versus the salinity level, distinguishing between configurations #1–#3 at $d_{UW}=$ 12 cm and configurations #4–#11 at $d_{UW}=$ 6 cm. Vertical error bars are added to highlight the uncertainty contributions in the RSSI measurements, while the positioning errors in $d_{UW}$ of ±0.5 cm are accounted for by plotting a range of values where the estimated RSSI can fall (triangular markers). The test-measured RSSI is obtained as the average of the SF-mean RSSI for each configuration.

These outcomes prove a good match between modeled and measured values even assuming a small positioning error, thus validating the proposed link budget model. Evidently, these results are strongly linked to the considered scenario and substantial difference can be observed moving from a controlled laboratory environment to a real scenario, where additional miscellaneous losses can contribute to the degradation of the received signal strength. This can be seen by comparing the results shown till now, gathered in laboratory conditions, with those obtained in the field in the experimental conditions described in Section 4.2. The results are reported in Table 3 for each SF, in terms of RSSI and SNR mean and standard deviation and PL percentage.

A larger variability in the measured data with respect to the laboratory outcomes despite similar depth and salinity conditions can be noticed both in the RSSI and in the SNR, leading also to a higher packet loss. This fact can be justified with the higher disturbances of the field deployment that add up to a higher positioning error of the transmitter, due for example to the presence of waves, that introduces subsequent increases and decreases of the $d_{UW}$ by few centimeters, and the tide, that causes a trend in the data due to the variation of the water level above the node.

**Figure 10:**
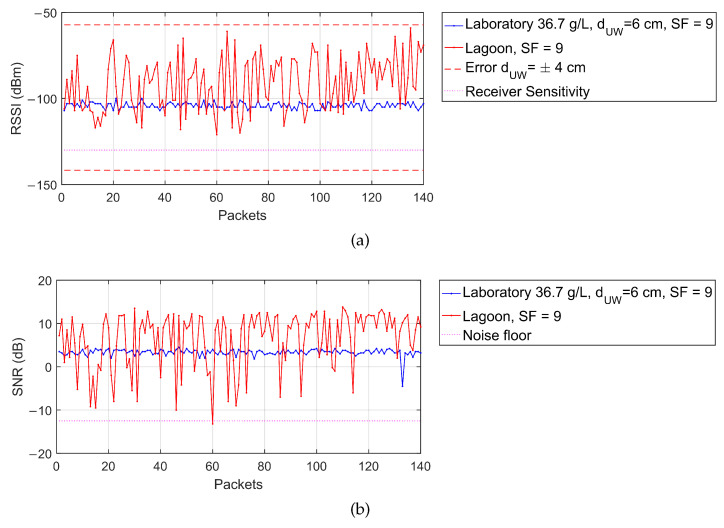
(**a**) RSSI and (**b**) SNR trends for SF 9 during field tests (red plots) and laboratory tests in configuration #9 (blue plots) together with the nominal receiver sensitivity and the noise floor levels for SF 9 (magenta lines) and the estimated RSSI according to Equation (27) assuming salinity of 35 g/L and $d_{UW}=6\pm 4$ cm (dashed red lines).

The waves variability can be better observed from Figure 10, where portions of the RSSI and SNR trends for SF 9 are reported in red, together with the analogous trends measured during the laboratory tests for SF 9 (in blue). Since an average salinity of 35 g/L and a depth $d_{UW}=6$ cm can be assumed for the lagoon test, the laboratory test configuration used as comparison is #9 (36.7 g/L and $d_{UW}=6$ cm). For clarity, both the nominal receiver sensitivity and the noise floor levels for SF 9 are added in magenta [45], along with the RSSI estimation according to Equation (27) assuming salinity of 35 g/L and $d_{UW}=6\pm 4$ cm (dashed red lines). It can be pointed out that the outcomes of the lagoon tests are quite consistent with the model-based prediction and with the assumed error of ±4 cm. Moreover, the proximity to the sensitivity and noise limits is the main reason determining the very high observed packet loss. Finally, from Figure 10 also the trend due to the tide can be appreciated, determining a drift in the RSSI of the lagoon tests, which was absent during the laboratory measurement campaign.

In conclusion, the performed experimental tests prove the feasibility of the LoRaWAN transmission with saline water from a transmitter node positioned just below the water surface. The critical context characterized by severe attenuation partially compromises the reliability of the transmission in operating conditions far from those in the laboratory. It is also true that, depending on the considered application, the high packet loss may not be a critical issue, for example in high-water or fill-level alarm systems advising just the crossing of a certain water point. Hence, according to the application, it is advisable to choose the best radio settings guaranteeing the optimum trade off between energy saving and reliability of the transmission, and the proposed link budget model can be a valid tool to orient this choice. Of course, the obtained results are very context-specific and by changing the test conditions, greater or smaller depths can be reached.

## 6. Conclusions

This paper proves the feasibility of the LoRaWAN transmission from a sensor node submerged in salt water at a depth of 6 cm, reinforcing the results already presented in [8] for the fresh water case and validating the usability of the LoRa technology in contexts of underwater to above water transmissions. Although the packet loss is minimal and the available link margin is sufficient in laboratory test conditions, the reliability of the transmission degrades in the case of field measurements where noise and loss factors increase. However, the results remain satisfactory as they demonstrate the usability of this technology in a set of applications based on transmitter nodes close to the water surface (e.g., systems for the monitoring of water sport athletes mounted in the swim cap, monitoring of bridges and harbor structures, water parameter acquisition) and where the reliability requirement is not mandatory (e.g., high-water or fill-level alarm devices). More generally, these outcomes open the way for salt water monitoring applications relying on low-cost and low-power commercial devices, in contrast with other underwater communication systems that are usually not suitable for the development of pervasive monitoring systems because more complex and expensive infrastructures are required.

A great accordance is proved between experimental and predicted data, derived according to the proposed link budget model based on the ITU documentation for the estimation of the salt water electric permittivity. Hence, this model can be effectively used as a valid tool to investigate the preliminary feasibility of the LoRa radio channel in different operational conditions, thus allowing to tune the radio settings on the basis of the obtained results and of the project constraints.

## Figures and Tables

**Table 1:** The operational conditions used during the laboratory tests.

	Depth $d_{UW}$ (cm)	Salinity (g/L)
#1	12	0
#2	12	10
#3	12	20
#4	6	20
#5	6	23.3
#6	6	26.7
#7	6	30
#8	6	33.3
#9	6	36.7
#10	6	40
#11	6	43.3

**Table 2:** Numerical results for the laboratory tests performed according to the configurations of Table 1: means and standard deviations for RSSI and SNR values and PLs percentages.

			#1	#2	#3	#4	#5	#6	#7	#8	#9	#10	#11
SF 7	RSSI	$\mu_{RSSI}$ (dBm)	−62	−100		−87	−89	−93	−96	−101	−104	−103	−110
		$\sigma_{RSSI}$ (dB)	2	3		3	1	1	1	1	2	1	1
	SNR (dB)	$\mu_{SNR}$	9.4	4.4		8.9	8.7	8.2	7	4.2	1.2	2.6	−2.8
		$\sigma_{SNR}$	1.1	2.2		1.1	1.1	0.8	0.7	0.9	1.0	0.6	0.6
	PL (%)		0.5	1.0	100.0	1.0	0.5	0.5	2.5	0.5	2.0	6.0	6.5
SF 8	RSSI	$\mu_{RSSI}$ (dBm)	−63	−100		−86	−89	−93	−97	−103	−103	−103	−109
		$\sigma_{RSSI}$ (dB)	2	2		1	1	2	1	3	2	1	1
	SNR (dB)	$\mu_{SNR}$	10.5	5.8		10.5	9.9	9.4	7.9	2.9	2.2	2.9	−2.8
		$\sigma_{SNR}$	1.5	1.7		1.4	1.2	1	0.9	2.8	1.4	0.6	0.8
	PL (%)		0.0	0.0	100.0	0.5	0.5	0.0	1.0	0.5	1.0	7.0	5.5
SF 9	RSSI	$\mu_{RSSI}$ (dBm)	−66	−100		−88	−91	−95	−99	−103	−104	−104	−109
		$\sigma_{RSSI}$ (dB)	3	2		2	2	3	2	2	2	2	1
	SNR (dB)	$\mu_{SNR}$	11.7	7.1		11.3	10.6	9.6	8.0	4.5	3.3	3.1	−2.2
		$\sigma_{SNR}$	1.7	1.6		1.5	1.3	1.1	2.0	0.7	0.8	0.9	1
	PL (%)		0.5	0.0	100.0	0.0	1.5	1.0	0.5	0.0	0.5	4.5	5.5
SF 10	RSSI	$\mu_{RSSI}$ (dBm)	−61	−101	−125	−84	−88	−91	−95	−101	−102	−102	−108
		$\sigma_{RSSI}$ (dB)	3	3	2	1	1	2	1	1	1	1	2
	SNR (dB)	$\mu_{SNR}$	10.5	3.7	−16.0	10.0	10.4	8.7	7.7	4.5	3.4	3.1	−2.2
		$\sigma_{SNR}$	1.9	2.0	0.6	1.5	1.4	1.3	0.9	1.3	0.5	1.2	1.2
	PL (%)		0.0	1.5	93.0	2.50	0.5	1.0	0.0	1.0	3.5	3.0	6.3
SF 11	RSSI	$\mu_{RSSI}$ (dBm)	−62	−100	−125	−84	−88	−90	−95	−101	−102	−104	−107
		$\sigma_{RSSI}$ (dB)	3	3	2	2	2	2	2	2	2	3	2
	SNR (dB)	$\mu_{SNR}$	11.6	5.5	−16.5	11.1	11.0	10.0	8.3	5.44	4.2	2.4	−0.9
		$\sigma_{SNR}$	1.5	1.9	1.0	1.2	1.0	0.9	0.7	0.7	0.4	1.5	0.9
	PL (%)		0.5	0.5	44.0	1.5	0.5	0.5	0.0	1.0	0.5	3.5	4.0
SF 12	RSSI	$\mu_{RSSI}$ (dBm)	−62	−102	−126	−84	−92	−90	−100	−102	−105	−103	−108
		$\sigma_{RSSI}$ (dB)	3	6	2	3	7	2	6	4	4	3	2
	SNR (dB)	$\mu_{SNR}$	10.5	4.8	−17.7	9.5	9.1	8.1	7.1	4.4	3.0	1.9	−1.0
		$\sigma_{SNR}$	1.3	2.6	1.8	1.0	1.5	0.9	0.8	1.4	0.7	2.0	2.0
	PL (%)		0.0	8.0	51.0	0.5	0.0	0.5	0.0	7.0	1.0	1.5	6.0

**Table 3:** Numerical results for the field tests in the Venice lagoon: means and standard deviations for RSSI and SNR values and PLs percentages.

		SF 7	SF 8	SF 9	SF 10	SF 11	SF 12
RSSI	$\mu_{RSSI}$ (dBm)	−97	−90	−93	−97	−99	−115
	$\sigma_{RSSI}$ (dB)	14	17	16	15	17	7
SNR (dB)	$\mu_{SNR}$	2.6	5.5	5.2	2.4	−1.7	−7.2
	$\sigma_{SNR}$	5.6	7.0	7.3	7.5	7.7	6.2
PL (%)		67	67.25	59.5	61.5	82.75	96.5

## Data Availability

Not applicable.

## References

[1] Gussen C.M., Diniz P.S., Campos M.L., Martins W.A., Costa F.M., Gois J.N. (2016). A survey of underwater wireless communication technologies. J. Commun. Inf. Syst..

[2] Campagnaro F., Francescon R., Casari P., Diamant R., Zorzi M. Multimodal underwater networks: Recent advances and a look ahead. Proceedings of the 12th International Conference on Underwater Networks & Systems.

[3] (2019). Semtech, LoRa and LoRaWAN: A Technical Overview, Semtech Corporation. https://lora-developers.semtech.com/uploads/documents/files/LoRa_and_LoRaWAN-A_Tech_Overview-Downloadable.pdf.

[4] Di Renzone G., Parrino S., Peruzzi G., Pozzebon A., Bertoni D. (2021). LoRaWAN underground to aboveground data transmission performances for different soil compositions. IEEE Trans. Instrum. Meas..

[5] Di Renzone G., Fort A., Mugnaini M., Parrino S., Peruzzi G., Pozzebon A. Interoperability among sub-GHz technologies for metallic assets tracking and monitoring. Proceedings of the 2020 IEEE International Workshop on Metrology for Industry 4.0 & IoT.

[6] Di Renzone G., Fort A., Mugnaini M., Peruzzi G., Pozzebon A., Vignoli V. LoRaWAN transmission system capability assessment in industrial environment under temperature and humidity characterization. Proceedings of the 2021 IEEE International Instrumentation and Measurement Technology Conference (I2MTC).

[7] Cappelli I., Fort A., Mugnaini M., Parrino S., Pozzebon A. Underwater to above water LoRa transmission: Technical issues and preliminary tests. Proceedings of the 24th IMEKO TC4 International Symposium and 22nd International Workshop on ADC and DAC.

[8] Cappelli I., Fort A., Mugnaini M., Parrino S., Pozzebon A. (2022). Underwater to above water LoRaWAN networking: Theoretical analysis and field tests. Measurement.

[9] Moore R.K. (1967). Radio communication in the sea. IEEE Spectr..

[10] Gabillard R., Degauque P., Wait J. (1971). Subsurface electromagnetic telecommunication—A review. IEEE Trans. Commun. Technol..

[11] Siegel M., King R. (1973). Electromagnetic propagation between antennas submerged in the ocean. IEEE Trans. Antennas Propag..

[12] Fattah S., Gani A., Ahmedy I., Idris M.Y.I., Targio Hashem I.A. (2020). A survey on underwater wireless sensor networks: Requirements, taxonomy, recent advances, and open research challenges. Sensors.

[13] Grosch A., Enneking C., Greda L.A., Tanajewski D., Grunwald G., Ciećko A. (2020). Theoretical concept for a mobile underwater radio-navigation system using pseudolite buoys. Remote Sens..

[14] Al-Shamma’a A.I., Shaw A., Saman S. (2004). Propagation of electromagnetic waves at MHz frequencies through seawater. IEEE Trans. Antennas Propag..

[15] Shaw A., Al-Shamma’a A.I., Wylie S.R., Toal D. Experimental investigations of electromagnetic wave propagation in seawater. Proceedings of the 2006 European Microwave Conference.

[16] Smolyaninov I., Balzano Q., Davis C.C., Young D. (2018). Surface wave based underwater radio communication. IEEE Antennas Wirel. Propag. Lett..

[17] Smolyaninov I., Balzano Q., Young D. (2020). Development of broadband underwater radio communication for application in unmanned underwater vehicles. J. Mar. Sci. Eng..

[18] Mendez H.F.G., Gac C., Le Pennec F., Person C. High performance underwater UHF radio antenna development. Proceedings of the Oceans 2011 IEEE-Spain.

[19] Shaneyfelt T., Joordens M.A., Nagothu k., Jamshidi M. RF communication between surface and underwater robotic swarms. Proceedings of the 2008 World Automation Congress.

[20] Nagothu K., Joordens M., Jamshidi M. Communications for Underwater Robotics Research Platforms. Proceedings of the 2008 2nd Annual IEEE Systems Conference.

[21] Lin K., Hao T., Zheng W., He W. Analysis of LoRa link quality for underwater wireless sensor networks: A semi-empirical study. Proceedings of the 2019 IEEE Asia-Pacific Microwave Conference (APMC).

[22] Santos M.O., Faria S.M.M., Fernandcs T.R. Real Time Underwater Radio Communications in Swimming Training Using Antenna Diversity. Proceedings of the 2021 Telecoms Conference (ConfTELE).

[23] Dala A., Arslan T. (2021). Design, implementation, and measurement procedure of underwater and water surface antenna for Lora communication. Sensors.

[24] Yoon H.S., Lee S.Y., Kim J.T., Yi J.H. (2012). Field implementation of wireless vibration sensing system for monitoring of harbor caisson breakwaters. Int. J. Distrib. Sens. Netw..

[25] Tronci E.M., Nagabuko S., Hieda H., Feng M.Q. (2022). Long-Range Low-Power Multi-Hop Wireless Sensor Network for Monitoring the Vibration Response of Long-Span Bridges. Sensors.

[26] Jalalifar S., Kashizadeh A., Mahmood I., Belford A., Drake N., Razmjou A., Asadnia M. (2022). A smart multi-sensor device to detect distress in swimmers. Sensors.

[27] Parri L., Parrino S., Peruzzi G., Pozzebon A. A LoRaWAN network infrastructure for the remote monitoring of offshore sea farms. Proceedings of the 2020 IEEE International Instrumentation and Measurement Technology Conference (I2MTC).

[28] Pule M., Yahya A., Chuma J. (2017). Wireless sensor networks: A survey on monitoring water quality. J. Appl. Res. Technol..

[29] Lin Y.P., Mukhtar H., Huang K.T., Petway J.R., Lin C.M., Chou C.F., Liao S.W. (2020). Real-time identification of irrigation water pollution sources and pathways with a wireless sensor network and blockchain framework. Sensors.

[30] Suzuki T., Kato K., Makihara E., Kobayashi T., Kono H., Sawai K., Kawabata K., Takemura F., Isomura N., Yamashiro H. (2014). Development of underwater monitoring wireless sensor network to support coral reef observation. Int. J. Distrib. Sens. Netw..

[31] Knight P., Bird C., Sinclair A., Higham J., Plater A. (2021). Testing an “IoT” tide gauge network for coastal monitoring. IoT.

[32] Sadiku M.N. (2014). Elements of Electromagnetics.

[33] Seybold J.S. (2005). Introduction to RF Propagation.

[34] Somaraju R., Trumpf J. (2006). Frequency, temperature and salinity variation of the permittivity of seawater. IEEE Trans. Antennas Propag..

[35] Hunt K.P., Niemeier J.J., Kruger A. RF communications in underwater wireless sensor networks. Proceedings of the 2010 IEEE International Conference on Electro/Information Technology.

[36] Hattab G., El-Tarhuni M., Al-Ali M., Joudeh T., Qaddoumi N. (2013). An under-water wireless sensor network with realistic radio frequency path loss model. Int. J. Distrib. Sens. Netw..

[37] International Telecommunication Union (ITU) (2021). Electrical Characteristics of the Surface of the Earth.

[38] Peres C., Pigeon M., Rather N., Gawade D.R., Buckley J., Jafarzadeh H., O’Flynn B. (2020). Theoretical models for underwater RFID and the impact of water salinity on the design of wireless systems. Int. J. Adv. Netw. Serv..

[39] Sun Z.H.I., Akyildiz I.F., Hancke G.P. (2011). Dynamic connectivity in wireless underground sensor networks. IEEE Trans. Wirel. Commun..

[40] Meneghello F., Campagnaro F., Diamant R., Casari P., Zorzi M. Design and evaluation of a low-cost acoustic chamber for underwater networking experiments. Proceedings of the 11th International Conference on Underwater Networks & Systems.

[41] Ghezzo M., Sarretta A., Sigovini M., Guerzoni S., Tagliapietra D., Umgiesser G. (2011). Modeling the inter-annual variability of salinity in the lagoon of Venice in relation to the water framework directive typologies. Ocean. Coast. Manag..

[42] Marine Biology in Chioggia, Parameters of Lagoon. https://chioggia.biologia.unipd.it/en/the-database/parameters-of-lagoon/.

[43] Semtech (2013). SX1272/3/6/7/8: LoRa Modem Designer’s Guide AN1200.13.

[44] Campagnaro F., Toffolo N., Pozzebon A., Francescon R., Barausse E., Airoldi L., Zorzi M. A Network Infrastructure for Monitoring Coastal Environments and Study Climate Changes in Marine Systems. Proceedings of the OCEANS 2022.

[45] Semtech (2019). SX1272/73: Datasheet.

